# The Legislature and the Bill

**Published:** 1893-12

**Authors:** 


### THE LEGISLATURE AND THE BILL.

We had hoped to announce to our readers this month the passage of the bill for the Board of Examiners. Up to this writing, however, final action has not been taken by the House of Representatives.

About the first of November the Judiciary Committee gave a courteous and patient audience to all those in any way interested in the bill. The regular profession was there unanimously asking for its passage. The eclectics and homeopaths, with their hired attorneys, were there to oppose it, along with the legal representative of the notorious quack and "robber," Dr. Flower, of Boston. A very sweet crowd, surely!

The appearance of the eclectics before the committee established the fact that they were not competent witnesses. Their opposition was weak, puerile and senseless. That of the homeopaths was somewhat better. However, the paper read by Dr. Orme was written on the false premise that the "regular" profession was seeking to gain an advantage for their school to the detriment of others. His statements were too general to constitute argument, but he did the best he could with a very poor subject. Concerning the speeches of the able and distinguished trio of lawyers, we do not think anything need be said, considering that their opposition was bought, and that if they had been paid a little more their sympathies and enthusiasm would have been enlisted in proportion on the other side. The speech of Col. Norwood, delivered in opposition, was of material assistance to the bill.

The present situation is somewhat complicated, from the fact that the committee appointed by the State Association introduced a new bill into the committee room, differing in some minor details from the one which passed the Senate at the last session. We fear that the sequel will show this to have been a mistake. The eclectics and homeopaths also introduced a bill providing that the board shall consist of five representatives from each school of medicine. We do not see how any just and intelligent body of men can adopt any such substitute.

The original bill, or the one drafted by the Assceiation Committee, should pass. The regular profession of the State will not be satisfied with any other.

---

Medical men, commonly called busy practitioners, are deeply indebted to the gentleman who furnishes the "Class Room Notes" for our esteemed contemporary, the *College and Clinical Record* (Philadelphia). For the most part these "notes" issue from the amphitheater of the Jefferson Medical College, and often contain bits of medical information startling in their originality, but practical and valuable nevertheless. For instance:

"Prof. Wilson thinks *Enteric Fever* often due to drinking contaminated water."

"Prof. Parvin says the *Mons Veneris* is very rarely the seat of disease."

"Prof. Keen says the first thing to do in a case of *Inflammation* is to remove the cause, if possible."

We trust that these eminent gentlemen are in possession of facts which warrant the above statements.

---

"*The local journal should be first and uppermost in every doctor's affections, and should have his good will and generous support.*

"*Contributions to its pages should be made often and brief; but it should be the bounden duty of every one to at least once a year make his offering in this channel.*

"*The subscription price of the local journal should never be allowed to run over, for there is never a time when the local journalist is not doing more for his profession than it is doing for him.*"—Dr. J. C. Culbertson, ex-editor of *Journal of American Medical Association.*

